# Ethical Considerations in the Design and Conduct of Clinical Trials of Artificial Intelligence

**DOI:** 10.1001/jamanetworkopen.2024.32482

**Published:** 2024-09-06

**Authors:** Alaa Youssef, Ariadne A. Nichol, Nicole Martinez-Martin, David B. Larson, Michael Abramoff, Risa M. Wolf, Danton Char

**Affiliations:** 1 Departments of Radiology, Stanford University School of Medicine, Stanford, California; 2 Center for Biomedical Ethics, Stanford University School of Medicine, Stanford, California; 3 Department of Psychiatry, Stanford University School of Medicine, Stanford, California; 4 Department of Ophthalmology and Visual Sciences, University of Iowa Hospital and Clinics, Iowa City; 5 Electrical and Computer Engineering, University of Iowa, Iowa City; 6 Division of Endocrinology, Department of Pediatrics, The Johns Hopkins School of Medicine, Baltimore, Maryland; 7 Department of Anesthesiology, Division of Pediatric Cardiac Anesthesia, Stanford, California

## Abstract

**Importance:**

Safe integration of artificial intelligence (AI) into clinical settings often requires randomized clinical trials (RCT) to compare AI efficacy with conventional care. Diabetic retinopathy (DR) screening is at the forefront of clinical AI applications, marked by the first US Food and Drug Administration (FDA) De Novo authorization for an autonomous AI for such use.

**Objective:**

To determine the generalizability of the 7 ethical research principles for clinical trials endorsed by the National Institute of Health (NIH), and identify ethical concerns unique to clinical trials of AI.

**Design, Setting, and Participants:**

This qualitative study included semistructured interviews conducted with 11 investigators engaged in the design and implementation of clinical trials of AI for DR screening from November 11, 2022, to February 20, 2023. The study was a collaboration with the ACCESS (AI for Children’s Diabetic Eye Exams) trial, the first clinical trial of autonomous AI in pediatrics. Participant recruitment initially utilized purposeful sampling, and later expanded with snowball sampling. Study methodology for analysis combined a deductive approach to explore investigators’ perspectives of the 7 ethical principles for clinical research endorsed by the NIH and an inductive approach to uncover the broader ethical considerations implementing clinical trials of AI within care delivery.

**Results:**

A total of 11 participants (mean [SD] age, 47.5 [12.0] years; 7 male [64%], 4 female [36%]; 3 Asian [27%], 8 White [73%]) were included, with diverse expertise in ethics, ophthalmology, translational medicine, biostatistics, and AI development. Key themes revealed several ethical challenges unique to clinical trials of AI. These themes included difficulties in measuring social value, establishing scientific validity, ensuring fair participant selection, evaluating risk-benefit ratios across various patient subgroups, and addressing the complexities inherent in the data use terms of informed consent.

**Conclusions and Relevance:**

This qualitative study identified practical ethical challenges that investigators need to consider and negotiate when conducting AI clinical trials, exemplified by the DR screening use-case. These considerations call for further guidance on where to focus empirical and normative ethical efforts to best support conduct clinical trials of AI and minimize unintended harm to trial participants.

## Key Points

**Question:**

How generalizable are current National Institutes of Health (NIH) ethical principles for conduct of clinical trials to clinical trials of artificial intelligence (AI), and what unique ethical considerations arise in trials of AI?

**Findings:**

In this qualitative study, interviews with 11 investigators involved in clinical trials of AI for diabetic retinopathy screening confirmed the applicability of current ethical principles but also identified unique challenges, including assessing social value, ensuring scientific validity, fair participant selection, evaluation of risk-to-benefit ratio in underrepresented groups, and navigating complex consent processes.

**Meaning:**

These results suggest ethical challenges unique to clinical trials of AI, which may provide important guidance for empirical and normative ethical efforts to enhance the conduct of AI clinical trials.

## Teaser

This qualitative study of investigators involved in clinical trials using artificial intelligence (AI) in diabetic retinopathy screening analyzes semi-structured interviews on the generalizability of National Institute of Health ethical research principles that may be unique to AI.

## Introduction

The integration of artificial intelligence (AI) into health care promises to address long-standing challenges, offering innovative solutions to improve patient outcomes, health equity, clinician productivity, and system efficiency.^1,2^ As the deployment of AI interventions expands, clinical evidence becomes increasingly crucial in validating their efficacy and safety.^3,4,5,6,7^ However, there exists a notable gap between the extensive theoretical research on ethical concerns in AI applications in health care and the practical challenges encountered by clinical investigators in clinical settings.^8^ This empirical study aims to bridge this gap by examining the practical ethical considerations in the design and implementation of clinical trials involving AI.

Early detection of diabetic retinopathy (DR) is a vanguard area in clinical AI; the first US Food and Drug Administration (FDA) De Novo–authorized autonomous AI was for diabetic eye examinations.^9^ We collaborated with investigators from the first National Institutes of Health (NIH)-funded randomized clinical trial (RCT) of autonomous AI, the AI for Children’s Diabetic Eye Exams Study (ACCESS), which was designed to determine the efficacy of autonomous AI screening for DR in a diverse population of youth with diabetes.^10^

Ethical frameworks for clinical research, shaped by landmark documents such as the Nuremberg Code, Declaration of Helsinki, Belmont Report, CIOMS guidelines, and the US Common Rule, form the bedrock of research ethics.^11,12,13^ Emanuel et al^12^ have further delineated 7 core principles for clinical trial ethics, endorsed by the NIH: social and clinical value, scientific validity, fair participant selection, favorable risk-benefit ratio, independent review, informed consent, and respect for human participants.^14^ However, the complexities inherent to AI, such as clinical efficacy, algorithmic fairness, and reproducibility of results, pose unique challenges.^15,16,17^ Systematic reviews have highlighted significant limitations in AI clinical trials, such as the absence of clinically relevant endpoints and a high risk of bias, raising questions about the suitability of traditional ethical frameworks in AI contexts.^4,18,19,20,21^ While there is a consensus on the necessity for increased transparency of randomized clinical trials of AI (AI-RCTs), current guidelines primarily focus on standardized reporting and fall short from addressing ethical considerations in the design of these clinical trials.^22,23^

This qualitative study aimed to address 2 primary research questions: (1) To what extent are the 7 NIH ethical principles^14^ created by Emanuel and Grady^12^ generalizable to clinical trials of AI? and (2) What are the ethical considerations that may be unique to clinical trials of AI?

## Methods

This qualitative study was approved by the Johns Hopkins Medicine institutional review board. All study participants were informed about the waiver of written consent and provided verbal consent to participate voluntarily, without financial compensation. We followed the Consolidated Criteria for Reporting Qualitative Research (COREQ) reporting guidelines.

### Study Design, Participants, and Recruitment

#### Study Design

We employed both a deductive and an inductive approach to data collection. We used a deductive approach described to test the applicability of the NIH’s 7 core ethical principles—clinical and social value, scientific validity, fair participant selection, favorable risk-benefit ratio, independent review, informed consent, and respect for human participants.^24^ We also utilized a modified grounded theory approach for the discovery of novel themes.^25^

#### Participants and Recruitment

Participants in this study included clinical investigators, institutional review ethicists, and clinical trialists involved in autonomous AI trials for diabetic retinopathy screening. The selection criteria were aligned with the study’s aim of examining ethical challenges in the design and conduct of AI-based clinical trials. Initially, purposive, nonprobabilistic sampling was used to recruit 6 participants from the ACCESS study (NCT05131451).^10^ Two authors (R.W. and D.C.) invited investigators from the ACCESS study to participate in this study. To enhance the generalizability of our findings, we employed snowball sampling method to identify participants involved in concurrent RCTs of AI for diabetic retinopathy screening in low-income countries, resulting in the inclusion of 3 participants from a nonprofit organization and 2 from the private sector.

### Data Collection and Analysis

Data collection occurred from November 2022 to February 2023. Interviews were conducted in English via video call (Zoom) by a qualitative research scientist (A.Y.) with over 7 years of experience in qualitative research. Interviews ranged from 30 to 60 minutes and were guided by a set of questions addressing demographic questions, the 7 ethical principles endorsed by the NIH—clinical and social value, scientific validity, fair participant selection, favorable risk-benefit ratio, independent review, informed consent, and respect for human participants—and open-ended questions to explore additional ethical considerations (eAppendix in Supplement 1). All interviews were transcribed by a professional service, and MAXQDA version 2022.2 software (VERBI GmbH) was used for managing and analyzing the data.

Following initial transcript analysis, a study coauthor (A.Y.) developed a codebook for systematic independent coding. Two authors (A.Y. and A.N.) independently coded all interviews, achieving a Cohen κ score greater than 0.8 for interrater reliability. Discrepancies were resolved through consensus coding with 3 coauthors (R.W., N.M., and D.C.). Theoretical saturation was achieved after the tenth interview, as no new insights emerged from the eleventh interview. To maintain reflexivity, we kept a detailed audit trail (A.Y.). This trail included reflections on both the interviewees’ and interviewer’s perceptions. These reflections were critically examined in weekly research meetings with all authors, challenging emerging hypotheses to reduce confirmation bias and ensure theme credibility.^26^

## Results

We conducted interviews with 11 investigators with experience conducting AI-RCT for DR screening, with a mean (SD) age of 47.5 (12.0) years (7 male [64%], 4 female [36%]; 3 Asian [27%], 8 White [73%]) (Table 1). Participants came from academia, the nonprofit sector, and industry, bringing diverse expertise in ethics, ophthalmology, translational medicine, biostatistics, AI development and deployment, and policy.

**Table 1.** Participants’ Demographic Characteristics

Participant No.	Affiliation	Race; ethnicity	Sex	Age, y	Medical practice experience, y	Research focus
1	Academia	White	Male	55-65	>25	Ophthalmology
2	Academia	White	Female	46-54	>10	Biostatistics/clinical trials
3	Academia	White	Male	55-65	>20	Informatics
4	Academia	Asian	Male	35-45	<10	Ophthalmology/ML
5	Nonprofit	White	Female	35-45	>10	AI implementation
6	Nonprofit	White	Male	55-65	>25	Ophthalmology
7	AI developer	White	Male	55-65	>25	AI developer/clinician-scientist
8	AI developer	White; non-Hispanic	Female	35-45	>10	Optometrist
9	Academia	Asian; non-Hispanic	Female	35-45	>10	Diabetic retinopathy
10	Industry	White; non-Hispanic	Male	35-45	>10	Diabetic retinopathy
11	Industry	Asian; non-Hispanic	Male	35-45	>10	AI/ML health care

Abbreviations: AI, artificial intelligence; ML, machine learning.

### Themes Overview

While recognizing the importance of the 7 ethical principles in AI clinical trials, participants identified unique ethical challenges specific to AI trials. These challenges demand a nuanced understanding of how to appropriately apply these principles in the context of AI clinical trials. Table 2 outlines participants’ perspectives on the 7 principles within the context of AI clinical trials. Table 3 presents novel ethical considerations that emerged from the inductive analysis, highlighting specific challenges faced during the implementation of these trials.

**Table 2.** Ethical Considerations for Applying the 7 Ethical Principles to Clinical Trials Involving AI

Ethical principle	Description	Illustrative quotes	Unresolved questions
Social value	Participants recognized that while AI can enhance clinical outcomes similar to non-AI trials, measuring the social value of AI is complex, with challenges in defining and quantifying its broader benefits	“Just because AI is better than humans that do certain things doesn’t mean if we incorporate AI it will necessarily improve the outcomes or the metrics that we’re interested in. So I think it’s very important in clinical trials involving AI to show that it improves outcomes. That’s a completely open question by now that we don’t know for the most part whether incorporating AI in clinical workflow improves outcome, so I think this is a very important question to answer.” (participant 4) “I think we should see what [social value] is from the patient’s perspective that would be beneficial and then identify and measure that potentially with a quantitative metric.” (participant 8) “Having to do a dilated eye exam is a whole thing in trying to access those services, but if you can provide that first level of screening really easily in a lot of different locations and make it really easy, it’s going to ultimately reduce disparities in the disease if it’s detected earlier.” (participant 2)	How should social value be defined and measured in clinical trials of AI?
Clinical value	Investigators recognized that while AI can enhance clinical outcomes similar to non-AI trials, measuring the social value of AI is complex, with challenges in defining and quantifying its broader impact.	“Just because AI is better than humans that do certain things doesn’t mean if we incorporate AI it will necessarily improve the outcomes or the metrics that we’re interested in. So I think it’s very important in clinical trials involving AI to show that it improves outcomes. That’s a completely open question by now that we don’t know for the most part whether incorporating AI in clinical workflow improves outcome, so I think this is a very important question to answer.” (participant 4)	If AI is a system intervention, do we assess clinical impact based on individual patient outcomes or a clinic level?
Scientific validity	AI introduces unique challenges in maintaining scientific validity. Unlike traditional drug or medical device trials, AI trials often require significant adjustments to workflow and may involve different standards of care that vary across settings. The approach to individual vs group outcomes was debated, with suggestions to focus more on average outcomes for groups to better capture the systemic impact of AI interventions.	“I think there are some issues with AI around scientific validity because it’s more of a systems intervention and to see whether individual randomization, you know, it’s hard, right, because for most studies we prefer individual randomization. I think another real problem with AI studies is what is the usual care component and does it make sense to compare to usual care when…usual care can vary tremendously now and what happens when all of a sudden a health system already has an AI, so is this competing, right. Is it comparing one AI to the existing AI?” (participant 1)	What constitutes ground truth on clinical trials of AI? How should AI interventions be measured against a criterion standard when the standard of care varies across deployment environments?
Fair participant selection	Participant selection in AI clinical trials emerged as a key concern, particularly for patients experiencing health disparities who may be disproportionately affected by systemic algorithmic biases.	“The recruitment process for the RCT should reflect the general public, considering ethnicity, gender, and other variables to achieve a representative target recruitment population.…And then once you have a target recruitment population that’s representative of the general public then you do your usual randomization.” (participant 4) “It’s tricky to ensure equitable access to AI screening, especially for those less likely to receive regular diabetes care. Monitoring the prevalence of diabetic retinopathy post-AI implementation and understanding the reasons behind disparities in screening rates and follow-up care are crucial.” (participant 2)	How to ensure fair participant selection given health care accessbility disparities? How to ensure fair participant selection when the population of focus is underserved?
Favorable risk-benefit ratio	Balancing the risk-benefit ratio in AI trials poses additional complexities. Participants discussed the challenges of assessing the unknown risks of AI interventions against known clinical conditions. The phased evaluation of AI, starting with safety and progressing to efficacy, was suggested as a method to systematically assess AI interventions.	“I think my biggest concern about AI is, as I’ve said at the outset, there’s the potential for AI not to lead to greater equity, but actually to exacerbate existing inequity because of the problems you just touched on, that if it’s not trained in a particular setting, that it may not have been relevant in that setting. And that’s the big risk, you know, of course we train these things to only work with images that come from super-expensive cameras or come from, you know, the best, most recent x-ray and CT and MRI machines and stuff, then you basically…I said we produced a Cadillac that literally can’t be driven on the roads of lower middle income country cities, I mean [it] becomes a real problem. So I think there’s an obligation to try to produce the tools openly, the ideal thing is to be producing an AI that is designed for use in low resource setting[s].” (participant 6)	How to determine intention-to-treatment effects when AI algorithms may not be trained on representative samples?
Informed consent	Informed consent in AI trials involves additional layers of complexity. Participants pointed out the necessity of transparent communication about the potential use of patient data by developers and the ethical considerations surrounding compensation for data use. Concerns were also raised about the current informed consent practices not being fully adapted to the specifics of digital health and AI.	“It’s very important to [our institution] that the local hospital still owns the images and those images belong to the local hospital, those are their patients and this is theirs. We do sometimes have licenses to continue to use those images, whether it be for training [institution]’s AI or if it’s to train, you know, a commercial partner’s AI, but that is all spelled out in the agreements that we set up ahead of the study or ahead of the data collection. But yeah, I mean I think [institution] from the beginning wants to ensure that, you know, the ownership of the images is still with the local partner, and then there is potential, you know, a license to use those images in a deidentified manner for training.” (participant 5)	Should patients be compensated or otherwise benefit from the use of their data in refining AI algorithms during clinical trials? How to balance the need to educate patients about AI potential harms when the algorithm risks are unknown for underrepresented groups?

Abbreviations: AI, artificial intelligence; CT, computed tomography; MRI, magnetic resonance imaging; RCT, randomized clinical trial.

**Table 3.** Novel Ethical Consideration for Conducting Clinical Trials of AI

Theme	Description	Illustrative quote
Whose values prevail in AI systems design and implementation?	The broadly defined term “value” in AI system design is subject to varied interpretations across different stakeholders such as health systems, clinicians, and patients, highlighting conflicts in priorities and concerns about the system’s inflexibility to adapt to individual patient needs	“So patient value can take all what people really, really, really care about which includes spiritual and religious issues which do not go into health services research considerations, don’t go into societal considerations, don’t go into…so there’s a lot of…so the very word value I would bet is not well-defined.” (participant 3) “Somebody who had very limited resources couldn’t afford to pay $60 a month for drops.…So, the decision that we make about how much quality we can afford needs to be made with respect to local economic scales.” (participant 6)
Can AI integration enhance clinical workflows without compromising patient safety?	Integrating AI into clinical workflows presents a significant tension between enhancing operational efficiency and the potential risk of complicating existing workflows, with unresolved questions about the actual improvement of clinical outcomes	“I think part of it, to not be able to do an RCT with an AI tool, would come down to just clinical workflow. If you’re trying to interject something into a workflow that’s already overloaded and pretty strapped.…I think that’s a limitation when it comes to implementing these because you have to set up, you know, both arms. You have to then set up multiple workflows and you’re already…you’re already trying to interject a new workflow where it hadn’t been before, which can be complicated.” (participant 8) “I think the clinical value is that there’s quite a bit of hype about AI and we know that for sure AI can do certain things better than humans in many different contexts, but just because AI is better than humans that do certain things doesn’t mean if we incorporate AI will it necessarily improve the outcomes or the metrics that we’re interested in. So I think it’s very important to in clinical trial involving AI to show that it improves outcomes. That’s a completely open question by now that we don’t know for the most part whether incorporating AI in clinical workflow improves outcome, so I think this is a very important question to answer. And you could only answer that using randomized clinical trial.” (participant 4)
How to balance the economic incentives with the ethical obligations to adopt effective AI interventions that can improve patients’ outcomes?	There is a profound conflict between the economic demands of conducting high-cost randomized clinical trials and the ethical imperative to make AI tools universally accessible, raising concerns about health care inequities, especially in lower-income settings	“If you don’t get creators and people like me and investors excited about the potential return, it will stop. That’s just the way it is. I was struggling as a developer, what is the balance between making…so [that] if you see that more access and better outcomes is good and you expect people to pay for that, how do you put a charge so that you don’t make a charge too high?” (participant 7) “Randomized control trials…there’s sort of an inherent assumption that they have to cost 40 million dollars. I just think that’s unethical. We have to come up with a way of delivering the kind of evidence, the high-quality evidence that can provide in a way that’s affordable for lower middle-income countries.” (participant 6)
What are the ethical implications of expanding DR screening without enhancing treatment access?	Expanding AI-driven diabetic retinopathy screening to underserved populations without improving access to treatment could create ethical dilemmas, potentially increasing rather than decreasing health disparities	“If someone’s not accessing services for diabetes in general regularly and not coming in for their well visits, …they’re not controlling their condition in the first place, they’re probably coming in less, they’re less likely to get the AI screening even if it’s available to them in their clinic and they’re more at risk for diabetic retinopathy because they’re not [accessing care].…If we’re trying to target and reduce those disparities, I think it would be important then to see where they’re falling off and why they’re not getting screened or are they getting screened and not going for follow-ups.” (participant 2) “I’ve been pleasantly surprised at how well the generalization has shown so far.…But I also think that if you only train for people from one small part of the world, then you would have a generalization problem.” (participant 10) “If the AI is efficacious, it’s an accurate diagnostic, but is this cost-effective if it costs a million dollars to run? Whether a system is willing to pay for such a product is another question.” (participant 11)

Abbreviations: AI, artificial intelligence; DR, diabetic retinopathy; RCT, randomized clinical trial.

#### Nuanced Considerations of the 7 Ethical Principles in AI Clinical Trials

When applied to clinical trials of AI in clinical settings, participants identified several unique applications of the 7 ethical principles. Common themes across principles included the added difficulty in accounting for equitable access to care and the need for transparency with patients.

##### Social and Clinical Value

Participants recognized AI’s potential to improve clinical outcomes, comparable with the potential outcomes of non-AI based RCTs (ie, drugs or medical devices). Specifically, in this RCT focused on DR, they perceived AI’s potential to reduce health disparities as a clear metric for social value. However, they expressed uncertainty defining and quantifying the social value of an AI intervention compared with its clinical benefits.

##### Scientific Validity

Participants recognized RCTs as a criterion standard for demonstrating clinical efficacy of the AI intervention. However, they identified unique challenges specific to AI RCTs. One participant questioned the appropriateness of prioritizing individualized outcome parameters—such as patient outcome—rather than outcomes for groups or populations. They noted the difficulty of comparing AI interventions with the variable criterion standard of usual care, which can differ significantly across clinical settings.

##### Participant Selection

Fair participant selection in AI clinical trials emerged as a significant topic, particularly regarding the accurate representation of the patient population of focus. Study participants highlighted the challenges in evaluating the efficacy of the AI intervention across patient subgroups, who are often affected by limited access to care and can be underrepresented in clinical trials. One participant pointed out the complexities of ensuring equitable access studying the impact of AI screening on patient groups that may access less regular diabetes screening and care.

##### Favorable Risk-Benefit Ratio

Participants recognized the complexity of balancing the risks and benefits of AI interventions across diverse patient groups. They noted the difficulty in estimating the harm-to-benefit ratio of AI interventions relative to the known risks of standard care, a challenge exacerbated by limited representation of patient groups facing health inequities in retrospective standalone studies of algorithm performance.

##### Informed Consent

Participants identified key ethical concerns presented in AI clinical trials, emphasizing the need for transparent communication about the risk and benefits of an AI intervention tailored to patients with varying levels of health literacy. They questioned whether patients fully understand the extent toward which their data might be used beyond the trial itself. Additionally, concerns were raised about the adequacy of current informed consent processes and institutional ethical review readiness to assess the risks and benefits of AI interventions.

#### Novel Ethical Considerations in Clinical Trials of AI

Participants highlighted additional ethical challenges beyond the established 7 ethical principles (Table 3). These included: (1) Whose values prevail in AI systems design and implementation? (2) Can AI integration enhance clinical workflows without compromising patient safety? (3) How to balance the economic incentives with the ethical obligations to adopt effective AI intervention that can improve patients’ outcomes? (4) What are the ethical implications of expanding DR screening without enhancing treatment access?

##### Whose Values Prevail in AI Systems Design and Implementation?

Participants critiqued the broadly defined term “value,” noting its varied interpretations across different stakeholders including health systems, clinicians, and patients. They questioned whose values are prioritized during the design and implementation of AI systems. Additionally, concerns were raised about AI’s adaptability to individual patient needs. For instance, while clinicians can adjust treatments to ensure affordability and effectiveness, AI systems may lack this flexibility due to their predefined operational parameters and potential downstream effects.

Said a participant who specializes in informatics, “So patient value can take all what people really, really, really care about, which includes spiritual and religious issues which do not go into health services research considerations, don’t go into societal considerations…so the very word value I would bet is not well-defined.” A participant with a research focus in ophthalmology pointed out that cost of care varied by community, “Somebody who had very limited resources couldn’t afford to pay $60 a month for drops.…So, the decision that we make about how much quality we can afford needs to be made with respect to local economic scales.”

##### Can AI Integration Enhance Clinical Workflows Without Compromising Patient Safety?

From the participants’ perspective, integrating AI tools into clinical workflows introduces a significant tension. There is a drive for AI to enhance clinical workflows, but this drive carries the inherent risk of complicating an already complex workflow, especially when the definitive clinical benefits of AI are still unclear. This tension is further amplified when contemplating potential risks to research participants during clinical trials. “I think part of it, to not be able to do an RCT with an AI tool, would come down to just clinical workflow,” said a participant working in optometry. “If you’re trying to interject something into a workflow that’s already overloaded and pretty strapped.…I think that’s a limitation when it comes to implementing these because you have to set up, you know, both arms. You have to then set up multiple workflows and you’re already…you’re already trying to interject a new workflow where it hadn’t been before, which can be complicated.”

“I think the clinical value is that there’s quite a bit of hype about AI and we know that for sure AI can do certain things better than humans in many different contexts, but just because AI is better than humans that do certain things doesn’t mean if we incorporate AI will it necessarily improve the outcomes or the metrics that we’re interested in,” said a respondent specializing in ophthalmology and machine learning. “So I think it’s very important to, in clinical trial[s] involving AI, to show that it improves outcomes. That’s a completely open question by now that we don’t know for the most part whether incorporating AI in clinical workflow improves outcome, so I think this is a very important question to answer. And you could only answer that using [a] randomized clinical trial.”

##### How to Balance the Economic Incentives With the Ethical Obligations to Adopt Effective AI Intervention That Can Improve Patients’ Outcomes?

Participants highlighted a critical tension in developing and validating AI tools in health care, balancing economic pressures with ethical imperatives. The ethical mandate to make these tools universally accessible clashes with the high costs associated with conducting RCTs, deemed the criterion standard for validation, particularly in the US and Europe. This economic challenge has prompted AI developers to shift RCTs to developing countries, raising concerns about potentially deepening health care inequities.

One participant who is both an AI developer and clinician-scientist expressed the dilemma facing AI developers and health systems: “If you don’t get creators and people like me and investors excited about the potential return, it will stop. That’s just the way it is. I was struggling as a developer, what is the balance between making…so if you see that more access and better outcomes is good and you expect people to pay for that, how do you put a charge so that you don’t make a charge too high?” The same participant also added insight on payment models for AI, suggesting a focus on health equity. “How should we be paying for AI?” he said. “If as a taxpayer or society you’re paying for something, then health equity should be the main guiding star.”

One participant in ophthalmology criticized the inherently high costs associated with RCTs: “Randomized control trials…there’s sort of an inherent assumption that they have to cost 40 million dollars. I just think that’s unethical. We have to come up with a way of delivering the kind of evidence, the high-quality evidence that can provide in a way that’s affordable for lower middle-income countries.”

Finally, the practical implications of AI were discussed. “If you’re improving the screening, then that in and of itself is sufficient to understand this is something that benefits patients,” said a participant working in AI and machine learning in health care. “Now whether a system is willing to pay for such an [AI solution], that’s a different question.”

##### What Are the Ethical Implications of Expanding DR Screening Without Enhancing Treatment Access?

A stated goal of AI is to broaden the reach of ophthalmology screening to populations currently underserved, thereby reducing their risk of blindness from DR. However, participants highlighted a complex array of clinical and ethical questions associated with this proposed use of AI. One concern is whether merely expanding access to DR screening without simultaneously improving access to treatment might create ethical dilemmas downstream.

“If someone’s not accessing services for diabetes in general regularly and not coming in for their well visits,…they’re not controlling their condition in the first place, they’re probably coming in less, they’re less likely to get the AI screening even if it’s available to them in their clinic and they’re more at risk for diabetic retinopathy because they’re not [accessing care],” said a researcher specializing in biostatistics and clinical trials. “If we’re trying to target and reduce those disparities, I think it would be important then to see where they’re falling off and why they’re not getting screened, or are they getting screened and not going for follow-ups.”

One participant emphasized the potential benefits of AI for the “bottom billion,” a uniquely underserved population, highlighting the slower arrival of such technologies to these groups. Another noted the generalization issues that can arise if AI models are trained on data from a narrow demographic. “I’ve been pleasantly surprised at how well the generalization has shown so far,” he said. “But I also think that if you only train for people from one small part of the world, then you would have a generalization problem.”

Finally, the cost-effectiveness of AI diagnostics was questioned, particularly in contexts where the financial burden might outweigh the clinical benefits. “If the AI is efficacious, it’s an accurate diagnostic, but is this cost-effective if it costs a million dollars to run?” asked one participant. “Whether a system is willing to pay for such a product is another question.”

## Discussion

This study is, to our knowledge, the first to explore the practical ethical considerations involved in designing and executing AI clinical trials. We draw on the experiences of investigators conducting the first NIH-funded RCT of an autonomous AI for DR screening, along with related trials. While we found consensus among stakeholders regarding generalizability of the NIH’s 7 ethical principles to clinical trials of AI, we identified important areas of uncertainty regarding social value, scientific validity, fair participant selection, favorable benefit-risk ratio, and informed consent (Table 2). Thematic analysis of participants’ experiences in DR screening trials across various settings also highlighted novel ethical considerations specific to AI clinical trials, independent of the 7 principles.

When discussing the 7 ethical principles, defining and measuring the social value of AI in clinical trials proved to be complex. Perspectives ranged from prioritizing patient views to measurable reduction in health care inequities. Moreover, participants struggled to generalize social value across trials due to its context-dependent nature and the lack of defined metrics to measure the social impact of AI interventions. While the clinical value of AI in improving patient outcomes is similar to that of traditional drug or device trials, evaluating AI introduces additional complexities as it functions as both a cognitive tool and a workflow intervention. Establishing a control, typically defined as the standard of care, can vary significantly across different clinical environments. This variability may limit the generalizability of AI interventions, as an AI system validated in one setting might not perform effectively in another setting.

In addition, the goal of using an AI to expand access for populations with limited access presents an ethical tension between the desirable social value of reducing access inequity with the limitations of an AI with biased training data (at least until better access for all groups is delivered) while outcomes are still being evaluated. For future trials, it will be crucial for researchers to clearly define the desired social value and establish specific, measurable outcomes that demonstrate the AI intervention efficacy achieving this value.

Participants also expressed nuanced concerns about ensuring a favorable benefit-to-risk ratio and obtaining true informed consent for AI interventions. While uncertainty is inherent in clinical research, balancing the unknown risks of AI screening against the known risks of untreated DR presented unique challenges. Interviewees noted that different population subgroups likely had different risk-benefit ratios when AI risks are compared with current screening methods or the absence of screening. Moreover, challenges in ensuring fair patient selection were noted, particularly in scenarios where patients facing health disparities are less likely to access care. Ensuring informed consent emerged as a significant challenge, particularly in communicating the benefits, risks, and data use terms for AI interventions to participants with varying levels of health literacy.

The exploration of novel ethical considerations in the context of AI clinical trials revealed several critical issues (Table 3). Participants expressed concerns about whose values are prioritized in the design and implementation of AI systems, emphasizing that current definitions fail to capture the diverse needs and priorities of all stakeholders, including patients, clinicians, and health systems. An ethical challenge is emerging in trials of AI around value capture; if a desired outcome of an AI tool is “value” (cost, labor, or access savings), who decides what outcome value is prioritized and how value savings are redistributed within a health care system or community is unknown.^27^ This uncertainty presents a crucial ethical knowledge gap in ensuring that AI trials are responsive to clinical contexts.

The integration of AI into clinical workflows also generated tension. RCT investigators aimed to evaluate AI effectiveness without compromising patient safety or disrupting established workflows with proven efficacy. Clinicians expressed concern that modifying care workflows to accommodate AI could unintentionally affect patient care or increase staff workload. Therefore, it is crucial to carefully assess the impact of AI on clinical workflows before implementation.

Participants also raised ethical concerns about using AI to improve access and equity without a clear financial incentive. They questioned what would incentivize a health system to invest in such technologies. Participants expressed concern around using AI to improve screening access in resource-constrained settings. For example, in the context of an AI clinical trial for DR screening, the AI system may be tested in a well-resourced clinical setting where patient can immediately receive follow-up treatment from an ophthalmologist if diagnosed with DR. However, if this AI system is later to be deployed in an underresourced setting, even if it accurately identifies patients needing treatment, the lack of access to follow-up care might prevent these patients from receiving the necessary interventions. This raises an ethical question: Is it appropriate to evaluate the AI’s efficacy in a controlled environment with follow-up care, knowing that in its clincial application, such care might be inaccessible?

Findings from this study suggest that the concept of equipoise—the ethical balance necessary in clinical trials—is more complex in AI interventions. AI, as a systems intervention, not only affects individual patient care but also integrates with and transforms health care workflows and system operations, complicating the evaluation of its effectiveness in clinical trials.

### Limitations

This study had several limitations. The participant pool was relatively small, comprising 11 individuals from 4 US academic institutions, and was restricted to investigators involved in AI clinical trials. While the deductive approach allowed us to systematically apply the 7 ethical principles to our data, it may inherently have limited the scope of conclusions by focusing on predefined frameworks. However, the inductive components of our study enabled us to explore novel insights and themes that emerged directly from the data, thereby enriching our understanding and identification of ethical issues beyond the initial framework. Although the theoretical concepts uncovered may be applicable to AI clinical trials in other areas, the scope of this study was limited to clinical trials of AI in diabetic retinopathy screening. Thus, the generalizability of these findings requires further validation in future studies. Furthermore, it is important to note that the 7 principles centered in this article have been criticized as being parochial, or at least Western-centric.^28,29^ The potential vulnerability of the 7 principles raised by this criticism is magnified as AI development for health care is occurring globally.

## Conclusions

This study addresses an important gap in practical understanding of how clinical investigators actually navigate ethical considerations arising with the design and conduct of AI clinical trials. It reveals a general consensus on the utility of NIH’s 7 ethical principles for clinical trials of AI but also important areas of uncertainty in social value, scientific validity, fair participant selection, favorable risk-benefit ratio, and informed consent. These findings highlight important considerations that should be addressed in future iterations of ethical guidance for AI trials. As Emanuel and Grady^12^ aptly noted, “Like a constitution, these requirements can be reinterpreted, refined, and revised.…Yet these requirements must all be considered and met to ensure that clinical research, wherever practiced, is ethical.”

## Notes

Supplement 1. **eAppendix.** Interview Guide

Supplement 2.
Data Sharing Statement
