# Supplementary material for: Ethical Considerations in the Design and Conduct of Clinical Trials of Artificial Intelligence
Source: JAMA Netw Open. 2024 Sep 6;7(9):e2432482. doi: 10.1001/jamanetworkopen.2024.32482 (PMC11380101; doi:10.1001/jamanetworkopen.2024.32482)
Supplement: Supplement 1. — eAppendix. Interview Guide [file jamanetwopen-e2432482-s001.pdf]

Supplemental Online Content

Youssef A, Nichol AA, Martinez N, et al. Ethical considerations in the design and conduct of clinical trials of artificial intelligence. *JAMA Netw Open.* 2024;7(9):e2432482. doi:10.1001/jamanetworkopen.2024.32482

eAppendix. Interview Guide

This supplemental material has been provided by the authors to give readers additional information about their work.

© 2024 Youssef A et al. *JAMA Network Open.*

eAppendix. Interview Guide

Questions:
Part 1: Demographic Data:
- Race/Ethnicity
• Age
• Sex
• Affiliation
• Years of Practice
- Research/Professional Training
• Research Focus (if any)

Part 2: NIH principles for designing an ethical clinical trial:
So, now I would like to shift our discussion towards considering clinical trials for such tools. NIH has put forward 7 principles they feel comprise an ethical clinical trial. I'd like to ask you about each of these areas, in light of your experience with AI. We would like to see how these 7 principles apply to AI clinical trial design, and if you were to identify other principles that may be important to consider.

1. What do you view as the **main social value(s**) of doing clinical trials of AI tools?
    a. What specific questions a clinical trial of AI needs to answer?
    b. What is the perceived social value of AI-tools and what is the value of doing these AI-trials?
2. What do you view as the **clinical value(s**) of doing clinical trials of AI tools?
    a. What specific questions a clinical trial of AI needs to answer?
    b. What is the perceived clinical value of AI-tools and what is the value of doing these AI-trials?

3. How do you ensure the study of AI tools is **scientifically valid**? For a novel tool like AI, what are you comparing AI to, (what is the "gold standard"), to establish scientific validity of the AI-tool?
    a. What are appropriate study endpoints for AI tools and how would you decide on them?

4. What should an RCT of an AI-OI tool look like (study design)?
    a. Is a randomized or blinded study of AI possible to achieve? If not, what rigorous or robust study approaches would you take to demonstrate AI's effectiveness?

5. How do you think about study sample recruitment for an AI-RCT?
    a. Do you distinguish between **subject selection** for AI training data versus data generated by the clinical trial?
    b. How do you reconcile needs to consider or mitigate bias in training data with fair subject selection in a clinical trial?

6. How would you define a favorable risk-benefit ratio for a clinical trial? What do you think should be a **favorable risk-benefit ratio** for clinical trials of AI tools?
    a. What is the best way to minimize the risks and inconvenience to research subjects, to maximize the potential benefits, and to determine that the potential benefits to individuals and society are proportionate to, or outweigh, the risks?

© 2024 Youssef A et al. *JAMA Network Open.*

7. For AI clinical trials, who should be the locus of **independent research review**?
    a. Who should have independent oversight of clinical trials to evaluate study design and patient recruitment?

8. What safeguards or measures could you implement to protect research volunteers? Can you think of any other additional mechanisms that could be established to protect research volunteers?Are evaluations by granting agencies, local institutional review boards (IRBs), and data and safety monitoring boards enough protections for AI? Are there particular or novel questions you think reviewers should ask about an AI trial?

9. Regarding **Informed consent**, since much is still unknown about AI tools, how would you recommend ensuring potential participants: (1) are accurately informed of the purpose, methods, risks, benefits, and alternatives to the AI research, (2) understand how this information relates to their own clinical situation, and (3) can make a voluntary decision about whether to participate?
    a. What are your thoughts on informed consent for patient data as part of training data sets to build AI tools?
    b. Do your thoughts about training data and clinical trial data differ? If so, how & why?

10. Much of **respect for potential and enrolled subjects** centers on respecting their privacy and keeping their private information confidential, how do you do this with AI tools?
    a. What do you recommend as ways for participants to change their mind, to decide that the research does not match their interests, and to withdraw without penalty from training data and/or from an AI clinical trial?

<u>*Additional Questions:*</u>
How would you plan to monitor participant welfare and potential adverse events?
What is the best way to inform them about what was learned from the research?
Of the issues we discussed, which do you view as most concerning?
What would you see as the best approach to studying clinical AI?

<u>*Closing:*</u>

Do you think there are any other principles when it comes to AI clinical trial design that you would recommend us to consider?
Do you have any other comments or think there are any questions I should have asked?
Thank you for providing such thoughtful responses. I'll stop the recording now

© 2024 Youssef A et al. *JAMA Network Open.*
